# Professionals’ Treatment Preferences in the Prodromal Phase of Parkinson’s Disease: A Discrete Choice Experiment

**DOI:** 10.3233/JPD-223208

**Published:** 2022-07-08

**Authors:** Lieneke van den Heuvel, Wibe Hoefsloot, Bart Post, Marjan J. Meinders, Bastiaan R. Bloem, Anne M. Stiggelbout, Janine A. van Til

**Affiliations:** a Radboud University Medical Center, Donders Institute for Brain, Cognition and Behaviour, Department of Neurology, Centre of Expertise for Parkinson & Movement Disorders, Nijmegen, the Netherlands; b Radboud University Medical Center, Radboud Institute for Health Sciences, Scientific Centre for Quality of Healthcare, Centre of Expertise for Parkinson & Movement Disorders, Nijmegen, the Netherlands; c Medical Decision Making, Department of Biomedical Data Sciences, Leiden University Medical Centre, Leiden, the Netherlands; d University of Twente, Department of Health Technology and Services Research, Technical Medical Center, Enschede, the Netherlands

**Keywords:** Decision making, disease-modifying treatment, Parkinson’s disease, prodromal PD, professional’s preference

## Abstract

**Background:**

In Parkinson’s disease (PD), several disease-modifying treatments are being tested in (pre-)clinical trials. To successfully implement such treatments, it is important to have insight into factors influencing the professionals’ decision to start disease-modifying treatments in persons who are in the prodromal stage of PD.

**Objective:**

We aim to identify factors that professionals deem important in deciding to a start disease-modifying treatment in the prodromal stage of PD.

**Methods:**

We used a discrete choice experiment (DCE) to elicit preferences of neurologists and last-year neurology residents regarding treatment in the prodromal phase of PD. The DCE contained 16 hypothetical choice sets in which participants were asked to choose between two treatment options. The presented attributes included treatment effect, risk of severe side-effects, risk of mild side-effects, route of administration, and annual costs.

**Results:**

We included 64 neurologists and 18 last year neurology residents. Participants attached most importance to treatment effect and to the risk of severe side-effects. Participants indicated that they would discuss one of the presented treatments in daily practice more often in persons with a high risk of being in the prodromal phase compared to those with a moderate risk. Other important factors for deciding to start treatment included the amount of evidence supporting the putative treatment effect, the preferences of the person in the prodromal phase, and the life expectancy.

**Conclusion:**

This study provides important insights in factors that influence decision making by professionals about starting treatment in the prodromal phase of PD.

## INTRODUCTION

Parkinson’s disease (PD) is a progressive neurodegenerative disease with a range of causes and clinical presentations [1]. Effective symptomatic treatment is widely used in clinical practice, but no effective treatment is available yet to slow down or postpone the onset of disease manifestations. Nevertheless, several treatments in this field are currently being tested in phase 2 and 3 trials [1–3]. Such treatments focus, for example, on α-synuclein proteostasis, mitochondrial function, calcium homeostasis, reducing iron overload, insulin signaling pathways, lysosomal function, oxidative stress, and/or neuroinflammation [1–3]. Apart from pharmacological interventions, there is also growing evidence that lifestyle factors such as exercise, or a Mediterranean diet, may be associated with a reduced risk of developing PD [4, 5].

As neurodegeneration likely begins many years before the diagnosis of PD is established based on clinically manifest motor signs, disease-modifying treatments would especially be beneficial in the early stages of PD [6, 7]. The International Parkinson and Movement Disorder Society Task Force defined three different stages of PD: preclinical PD, prodromal PD, and clinical PD [8]. In the preclinical stage, neurodegeneration has started, but no clinical symptoms are present. In the prodromal stage of PD, neurodegeneration has started and symptoms and signs are present, but it is not yet possible to diagnose PD [8]. Diagnosing such persons with prodromal PD would allow for earlier treatment and thereby potentially slow down or even stop neurodegeneration, and thereby postpone or even prevent clinically manifesting PD from developing [9]. Criteria have been developed to determine the risk of a person being in the prodromal phase of PD, based on risk factors and diagnostic markers [10]. These criteria are currently used for research purposes only, and they provide an estimate of the risk of being in the prodromal phase of PD, but there is no absolute certainty that individuals will develop PD.

Not knowing whether a person who meets the criteria for prodromal PD will actually develop the disease makes the choice of prescribing a disease-modifying treatment challenging. The decision process associated with installing a putative disease-modifying treatment in persons who do not have the disease (yet) might well be different from the decision process that clinicians are more accustomed to, namely in making the decision to prescribe a specific symptomatic treatment for clinically discernible parkinsonian symptoms in a person diagnosed with PD. The decision to treat someone who carries a risk but is still asymptomatic involves balancing the unknown benefits with expected harms. To date, no research on professionals’ preferences regarding preventive treatment has focused on the prodromal phase of PD. When such disease-modifying treatments would become available, then the value that professionals assign to key information about potential preventive treatment could provide us first insight in what type of information and what cut-off values have an impact on decision making. Such information will help to understand the clinical reasoning of physicians, when it comes to the implementation in clinical practice. The aim of this study was to explore the importance that professionals assign to five key characteristics (treatment effect, risk of mild side-effects, risk of severe side-effects, route of administration, and annual costs) of putative disease-modifying treatment in the prodromal stage of PD. A second aim was to explore the likelihood of actually prescribing a disease-modifying treatment to persons in the prodromal stage of PD.

## METHODS

### Study population and recruitment

Eligible participants included Dutch neurologists and neurology residents in the 6^th^ and final year of their training. To recruit participants, an invitation was published in the online newsletter and sent to the e-mail membership list of the Movement Disorders workgroup of the Dutch Society for Neurology (Nederlandse Vereniging voor Neurologie), together reaching approximately 1450 members. The invitation contained an introduction to the study with a link to the survey. To increase response rate, a reminder was sent after three months to 70 neurologists and final year neurology residents in the researchers’ (BP and BB) professional network. Inclusion started in December 2020 and finished in May 2021.

**Fig. 1:**
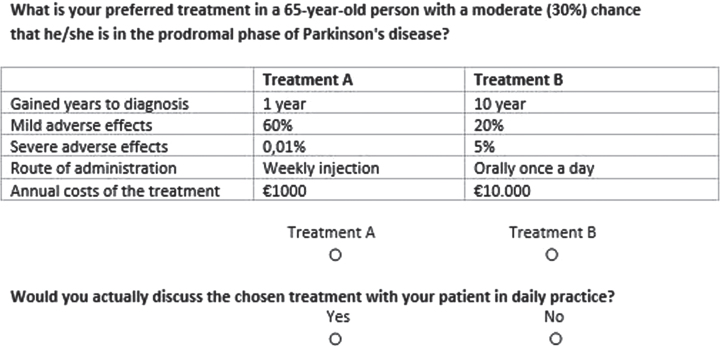
Example of a choice set presented in the discrete choice experiment.

**Table 1:** Attributes and levels for choosing a disease-modifying treatment for a person in the prodromal phase of Parkinson’s disease

Attributes	Level 1	Level 2	Level 3	Level 4
Number of years gained until to diagnosis	20 years	10 years	5 years	1 year
Risk of mild side effects	20%	40%	60%
Risk of severe side effects	0,01%	0,1%	1%	5%
Route of administration	Orally once a day	Orally three times a day	Weekly injection	Six-monthly injection
Annual costs	€ 100	€ 1,000	€ 10,000

### Study design

Preferences regarding treatment in the prodromal phase in PD were elicited in an online survey containing a Discrete Choice Experiment (DCE). DCEs, grounded in consumer theory and psychology of choice, are used in health care to examine respondents’ preferences for different healthcare interventions [11, 12]. This method is particularly useful in providing quantitative data in scenarios where no revealed preferences yet exist and therefore could guide further policy making and development. In a DCE, respondents answer multiple preference elicitation tasks. In each task, respondents are asked to indicate their preference for one of two or more hypothetical profiles, which are descriptions of possible interventions. Profiles consist of different attributes, which are the characteristics of the interventions that are compared (e.g., risk on side-effects), and levels, which are descriptions of the range of potential outcomes for each attribute (e.g., 5% chance of a side-effect). In the analysis, the extent to which the attributes of the intervention drive preferences, and the trade-offs respondents are willing to make between different attribute outcomes, can be assessed. For this study, an existing checklist for designing a DCE was used and provided in the Supplementary Material [13].

### Selection of attributes and levels

Attributes of hypothetical disease-modifying treatment in the prodromal phase of PD were selected based on a review of the literature and semi-structured interviews with six neurologists (Supplementary Methods and Results). The literature on generic treatment attributes used in DCEs was used for the initial attribute identification. Subsequently, interviews with neurologists were performed to determine how these identified treatment attributes would relate to hypothetical treatments in the prodromal phase of PD. Results from the interviews were used to select the final attributes (Table 1). Improvement in health in the prodromal phase of PD was defined as slowing down disease progression, which was operationalized into the attribute ‘delay in years to diagnosis’. Risk of side-effects was operationalized in two different attributes: risk of mild side-effects and risk of severe side-effects. Cost of treatment was defined as annual costs. Based on the interviews, a fifth treatment attributed was added: route of administration. For each attribute, 3 or 4 levels were defined, based on levels presented in the literature and information derived from the interviews. Final treatment attributes and levels were determined in a consensus meeting with the research team.

All interviewees mentioned the risk of the person being in the prodromal phase of PD as an important factor that would influence their decision-making. To study the effect of the risk profile on professionals’ preferences, the DCE was performed twice: one for a hypothetical person who had a high risk of being in the prodromal phase of PD (80%) and one for a hypothetical person who had a moderate risk (30%) (risk profiles were based on group consensus).

### Survey design

The survey consisted of three parts and was programmed using LimeSurvey, an online survey tool (Limesurvey GmbH, Hamburg, Germany). The first part of the survey contained an introduction with the overall context of the study. This was followed by socio-demographical questions, including age, gender, type of hospital they work at (academic or non-academic), number of years working experience, focus area within neurology, number of PD patients that they saw every month, and how familiar they were with research on the prodromal phaseof PD.

The second part of the survey contained the DCE. The section started with an explanation of the preference elicitation task, the definitions of the attributes and a definition of a high (80%) and moderate (30%) risk profile. Each choice set consisted of two full treatment profiles which included levels for all attributes (treatment A and treatment B). Respondents were asked to indicate their preference for either of the two treatments. Respondents were forced to answer each question, and no opt-out or status quo was given. After each choice set however, a dual response opt-out question was added: ‘’*Would you actually discuss the chosen treatment with your patient in daily practice? Yes or No*”.

The experimental design for the DCE was designed using the software R. First, the full factorial design was estimated. Then, choice tasks with dominant profiles of overlapping attribute-levels were removed from the full set. No restrictions on implausible attribute-level combinations were set. Then, 1000 random sets of 32 choice tasks were drawn from the full set, and the set with the highest D-efficiency was selected. This resulted in a design that was nearly balanced and nearly orthogonal. To reduce cognitive difficulty, the design was blocked into four blocks of eight choice sets. Questions within each block were randomized. Each respondent was randomized to receive one block of choice sets for a high risk profile and a different block of choice sets for a moderate risk profile. Thus, each respondent was asked to choose their preferred treatment for in total 16 choice sets.

An internal validity question was included, with a clear superior (maximum effect, minimal risk of side-effects, and minimal costs) and one inferior treatment choice (minimal effect, maximal risk of side-effects, and maximal costs).

The third part of the survey contained two qualifying questions. The first question was a multiple-option question on other important attributes that would influence the participant’s decision to start an intervention in the prodromal phase of PD. Answering options included: life expectancy, preference of the person in the prodromal phase, prodromal PD symptoms, expected treatment compliance, current lifestyle of the person in the prodromal phase, amount of evidence for the effect of the treatment, the extent of monitoring needed and ‘other’, in which the participant could fill out other aspects to consider. The second question was a multiple choice question on the participant’s opinion on lifestyle advice in the prodromal phase of PD (Supplementary Table 4).

The DCE was piloted in a face-to-face interview with eight neurologists to ensure comprehension, ease of use and completeness of the survey.

### Sample size

Different methods exist to calculate sample size in DCEs. A rule of thumb to calculate the required sample size is: *N*> $\frac{500\times \mathrm{largest}\;\mathrm{number}\;\mathrm{of}\;\mathrm{levels}}{\mathrm{number}\;\mathrm{of}\;\mathrm{choice}\;\mathrm{sets}\;\times \mathrm{number}\;\mathrm{of}\;\mathrm{alternatives}}$ [14].

Withthe proposed DCE characteristics, the required sample size was 63 according to this rule of thumb. However, as others propose at least 100 respondents [14], we aimed for a sample size between 63 and 100.

### Statistical analysis

Descriptive statistics were used to summarize the characteristics of the study sample. Only those participants who selected the dominant treatment option in the internal validity question, were included in the analysis. The choice sets were analyzed using an effect-coded conditional logit model [15] using Cox regression in SPSS Statistics 25 (IBM). The utility of the treatment alternatives was calculated by the sum of the β-coefficients of the corresponding attributes plus the error term [15–17]. The estimated β-coefficient is a preference weight, indicating the impact of that attribute on choosing a specific treatment. It represents the relative contribution of the attribute level to the utility that respondents assign to an alternative (part-worth utility estimate). Because effect-coding was used, the estimated preference weights for the hypothetical treatment attribute are relative to the mean effect, normalized at zero. The signs of the β-coefficients indicate whether the attribute has a negative or positive effect on utility. Differences between part-worth utility for different levels indicate the relative importance of moving from one level of an attribute to an adjacent level of that attribute: the greater the difference, the more important the change from one level t o the next (within-attribute). The relative importance of each attribute (between-attribute) is a measure of the relative influence of the change from worst to best outcome on each attribute on the overall utility of the treatment. Thus, it is a measure of the relative importance of the attribute in choosing between treatments. The relative importance of each attribute is calculated by taking the range in part-worth utility estimate for the best and worst levels of each attribute, and divide it by the sum of the range in part-worth utility of all other attributes [17]. Predefined subgroup analysis included analysis from data from neurologists only (Supplementary Tables 1 and 2).

A logistic regression model was used to analyze how individual treatment attributes and risk profiles influenced the choice to discuss the treatment with the person in the prodromal phase in daily practice, using only the chosen treatment from each choice set. The dependent variable was the choice: opt-in (yes) or opt-out (no). Independent variables included the treatment attributes and levels, and the risk profile.

The marginal rate of substitution represents the rate at which respondents are willing to trade off among the attributes. We calculated the willingness to accept an increase in risk of severe side-effects between the interval of 1–5% to gain an additional year in time to diagnosis, the two most highly valued attributes. The willingness to accept an increase in risk of severe side-effects to gain an additional life year in time to diagnosis was calculated by dividing the utility gain for gaining 1 additional year in time to diagnosis by the utility loss of 1% increased risk of severe side-effects. We calculated the utility loss from of an additional 1% risk of severe side-effects between the levels 1% and 5%, because the difference in utility in this attribute was greatest between those levels. We separately calculated the utility gain for gaining 1 additional year in time to diagnoses between the levels 1 and 5 year (i.e., the risk of severe side-effects participants were willing to accept when treatment effect increases from gaining 1 year to 2 years, from 2 to 3 years, 3 to 4 years, and 4 to 5 years) and similarly between 5 and 10 years, and 10 and 20 years.

### Ethical statement

The study protocol was approved by the Medical Ethics Committee of the Radboud university medical center and registered as 2020–6627. All participants gave online informed consent prior to thestudy.

## RESULTS

### Sample characteristics

In total, 138 people responded to the study invitation. From these, fifty-six respondents were excluded, due to incomplete surveys (*n* = 50), not meeting the inclusion criteria (*n* = 5), or an inconsistent answer to the internal validity question (*n* = 1). This resulted in a sample of 82 eligible respondents (63 neurologists and 19 last year neurology residents), included in the analysis (Table 2).

**Table 2:** Demographics of the professionals included in the analysis

Demographics
Number of participants	82
Gender (*n* (%) men)	35 (43%)
Age in years (mean (SD))	42.0 (9.3)
Working experience in current profession in years (mean (SD))	9.3 (6.9)
Number of PD patients per month (mean, (SD))	28.6 (36.9)
Profession (*n* (%))
Neurologist	63 (77%)
Neurology resident	19 (23%)
Hospital type (*n* (%))
Academic	22 (25%)
Non-academic	66 (75%)
Area of expertise includes movement disorders (*n* (%) yes)	56 (68%)
How familiar with developments prodromal PD (*n* (%))^*^
Extremely familiar	1 (1.2%)
Very familiar	7 (8.5%)
Moderately familiar	23 (28.0%)
Slightly familiar	35 (42.7%)
Not familiar at all	16 (19.5%)

^*^Participants were asked how familiar they feel with scientific developments in the field of prodromal PD.

**Table 3:** Estimated part-worth utility coefficients for the attribute levels

	High risk (80%)					Moderate risk (30%)				
	β	SE	*p*	Exp(β)	95% CI Exp(β)	β	SE	*p*	Exp(β)	95% CI Exp(β)
Effect			0.000					0.000
20 years gained	1.888	0.288	0.000	6.605	3.759–11.608	1.092	0.163	0.000	2.979	2.163–4.103
10 years gained	1.069	0.160	0.000	2.913	2.131–3.982	0.815	0.126	0.000	2.258	1.763–2.892
5 years gained	–0.262	0.191	0.171	0.770	0.529–1.119	–0.158	0.145	0.277	0.854	0.642–1.135
1 year gained	–2.695	0.443	0.000	0.068	0.028–0.161	–1.748	0.209	0.000	0.174	0.116–0.262
Mild side-effects			0.028					0.000
20% risk	0.493	0.200	0.014	1.637	1.105–2.424	0.567	0.155	0.000	1.763	1.302–2.387
40% risk	–0.097	0.156	0.535	0.908	0.669–1.232	–0.101	0.126	0.422	0.904	0.706–1.157
60% risk	–0.396	0.165	0.017	0.673	0.487–0.931	–0.466	0.125	0.000	0.628	0.491–0.803
Severe side-effects			0.000					0.000
0.01% risk	0.954	0.252	0.000	2.597	1.586–4.251	0.804	0.170	0.000	2.234	1.602–3.115
0.1% risk	0.762	0.278	0.006	2.142	1.242–3.692	0.431	0.160	0.007	1.539	1.125–2.107
1% risk	0.013	0.174	0.941	1.013	0.720–1.426	0.097	0.132	0.465	1.101	0.850–1.427
5% risk	–1.729	0.326	0.000	0.178	0.094–0.336	–1.332	0.178	0.000	0.264	0.186–0.374
Route of administration			0.004					0.023
Orally daily	0.124	0.339	0.713	1.132	0.583–2.199	–0.335	0.197	0.089	0.715	0.487–1.052
Orally 3 times a day	–0.194	0.252	0.442	0.824	0.502–1.351	–0.049	0.152	0.749	0.952	0.706–1.284
Weekly injection	–0.370	0.140	0.008	0.690	0.525–0.908	–0.076	0.125	0.541	0.927	0.726–1.183
Six-monthly injection	0.440	0.210	0.036	1.553	1.030–2.342	0.460	0.151	0.002	1.584	1.179–2.129
Annual costs			0.000					0.000
€ 100	0.650	0.202	0.001	1.915	1.289–2.847	0.390	0.121	0.001	1.477	1.164–1.974
€ 1000	0.221	0.127	0.081	1.248	0.973–1.600	0.157	0.110	0.153	1.171	0.943–1.453
€ 10.000	–0.871	0.212	0.000	0.418	0.276–0.634	–0.547	0.117	0.000	0.579	0.460–0.728

The signs of the β-coefficients indicate whether the attribute has a negative or positive effect on utility. SE, standard error.; CI, confidence interval.

### Relative importance of treatment attributes

**Fig.2:**
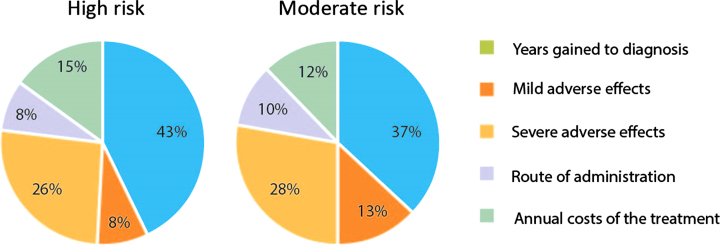
Relative importance of attributes for choosing a treatment, for a person with a high risk (80%) and a moderate risk (30%) on being in the prodromal phase of Parkinson’s disease.

The estimated β-coefficients for the attribute levels are presented in Table 3. The preferred order of importance for attribute levels was as expected, with higher preference for higher effectiveness, lower risk of side-effects, longer administration intervals, at minimal costs. In both risk profiles, the most important attribute of treatment (between-attributes, Fig. 2) was the increase in ‘*years gained to diagnosis*’, followed by the decrease in the ‘*risk of severe side-effects*’. Participants attached lower importance to the attributes ‘*risk of mild side-effects*’, ‘*route of administration*’ and ‘*annual costs*’. Subgroup analysis, excluding the data from residents, showed comparable results Supplementary Tables 1 and 2).

**Fig. 3:**
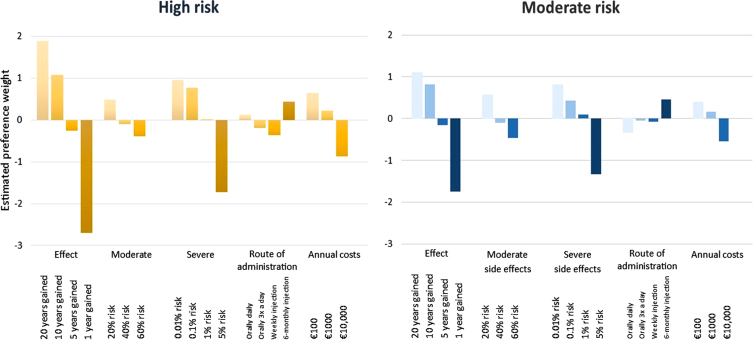
Relative contribution of the attribute level on choosing treatment. Data represents the estimated preference weights as presented in Table 3.

The increase in overall value of the treatment as a result of moving from one level to the subsequent level within one attribute was highest if effectiveness increased from ‘*1 year gained to diagnosis*’ to ‘*5 years gained to diagnosis*’, followed by a decrease in ‘*risk of severe side-effects*’ from 5% to 1% (Table 3 and Fig. 3). This was the case in both a moderate risk profile (difference in estimated β-coefficient of 1.59 for ‘*years gained to diagnosis*’, and 1.43 for ‘*risk of severe side-effects*’), as for a high risk profile (difference of 2.43 for ‘*years gained to diagnosis*’, and 1.74 for ‘*risk of severe side-effects*’).

### Marginal rate of substitution

We calculated the willingness to accept an increase in risk of severe side-effects (between the interval of 1–5% risk) to gain an additional disease-free year in time to diagnosis, which were the two most important attributes. For persons with a high risk profile, participants were willing to accept a 1.4% increase in risk of severe side-effects to gain one additional year in time to diagnosis between gaining years 1 and 5. Willingness to accept risks decreased with life years gained beyond 5 years. Participants were willing to accept a 0.6% increase in risk to gain an additional year between years 5 and 10, and a 0.2% increase in risk to gain an additional year between years 10 and 20. In persons with a moderate risk profile, participants were willing to accept a 1.1% increase in risk of severe side-effects to gain an additional year in time to diagnosis between gaining years 1 and 5, a 0.5% increase in risk to gain an additional year between years 5 and 10, and a 0.1% increase in risk to gain an additional year between years 10 and 20.

### Choosing treatment in daily practice

**Fig. 4:**
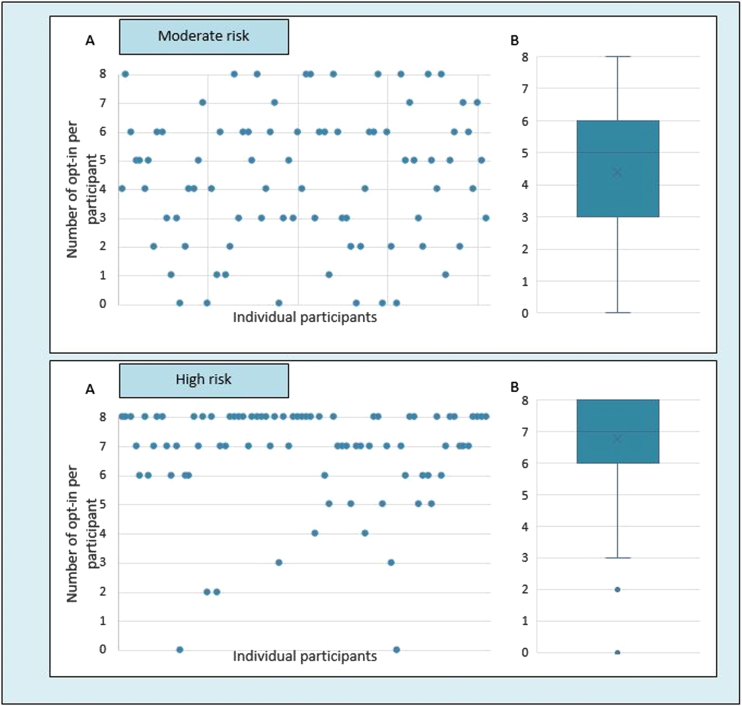
Number of times participants indicated that they would discuss the chosen treatment in daily practice. Scatterplot (A) and boxplot (B) presenting the number of times each individual participant indicated that they would discuss the chosen treatment in daily practice (opt-in) for a patient with a moderate risk (30%) and a high risk (80%) of being in the prodromal phase of PD. Each participant completed eight choice sets for each risk profile.

After each choice set, participants were asked whether they would discuss the preferred treatment with an actual person who is in the prodromal phase. Participants were more likely to discuss the preferred treatment with persons with a high risk profile compared to a moderate risk profile (Fig. 4). For persons with a moderate risk profile, respondents showed more variation in their likelihood to discuss treatment than in persons with the high risk profile. The decision to discuss treatment was primarily driven by the risk profile and less by the attribute levels Supplementary Table 3).

## Other factors

When asked which other factors would influence participants’ treatment choice, most participants chose ‘*amount of evidence supporting treatment effect*’ (92.8% of the participants), ‘*preference of the person in the prodromal phase*’ (90.4%), and ‘*life expectancy*’ (89.2%). Less frequently chosen were ‘*expected treatment compliance*’ (59.0%), ‘*extent of monitoring needed*’ (44.6%), '*symptoms that are already present'* (33.7%), ‘*lifestyle*’ (24.1%). Additional factors mentioned in the open text included comorbidity (*n* = 2), cost-effectiveness (*n* = 2), number needed to treat (*n* = 1), long term side-effects (*n* = 1), family history of PD (*n* = 1), possibility to prevent serious side-effects with monitoring (*n* = 1), reversibility of side-effects (*n* = 1), partner support (*n* = 1) and insurance coverage (*n* = 1).

## DISCUSSION

In this study, we elicited which factors will influence hypothetical decision-making by professionals about starting an intervention in the prodromal phase of PD. Treatment effect and the risk of severe side-effects were valued as most important when choosing a putative disease-modifying treatment, for both persons with a moderate risk and for those with a high risk of being in the prodromal phase PD. The lack of difference between both risk profiles is surprising. We hypothesized that professionals would value the risk of severe side-effects as more important in persons with a moderate risk profile compared to persons with a high risk profile, and the treatment effect as more important in persons with a high risk profile compared to a moderate risk profile. Participants did, indeed, indicate that they would discuss the treatment in daily practice more often in persons with a high risk profile. This might indicate that the prodromal risk profile does not necessarily influence *which* treatment professionals prefer, but that it does influence *if* they would actually choose atreatment.

Most participants (92.8%) chose the amount of evidence for the effect of the treatment as an additional important factor influencing their treatment choice. When disease-modifying treatments would become available, sufficient evidence-based information on treatment effect should thus be provided to address this need. Another frequently chosen factor that would influence treatment choice was the preference of the person in the prodromal phase. This highlights the dominant role of the person who carries the risk of developing PD, and that treatment choice is not a decision made solely by the professional. Literature shows that the attitude of professionals and patients on treatment attributes might differ. For example, a study that was performed among both professionals as well as patients, focused on factors involved in choosing treatment for preventing heart attacks [18]. They found that professionals often accept smaller benefits of preventive treatment than expected by the patients. It is unknown which values and priorities persons with prodromal PD themselves have when choosing whether or not to start a putative disease-modifying treatment. A study that evaluated the perspective of persons with PD on early diagnosis found that the majority indicated that they would have liked to know their risk of developing PD, but only if they would have received instructions on how lifestyle changes may alter the course of the disease [19].

The process of decision-making in PD is more complex than only weighing potential treatment characteristics, from a physician’s perspective. To judge the benefits of an early diagnosis in the prodromal phase, e.g., one should include the patient’s perspective as well, acknowledging that this is not a one-size-fits-all decision. Some people will want to know their risk, whether or not there are neuroprotective strategies, while others would rather never know if they are at risk. Finding the right timing of an early diagnosis takes an understanding of the person’s life-goals, fears, potential benefits and harms [20]. Risk disclosure in the prodromal phase should also should consider the risk profile of an individual. For example, the MDS research criteria for prodromal PD present a positive likelihood ratio of 130 for individuals with idiopathic, polysomnographic-proven REM sleep behavior disorder (RBD), while people who experience constipation present only a positive likelihood ratio of 2,5 [10]. When the likelihood of becoming diagnosed with PD is low and when it comes with high uncertainty in the risk calculations, clinicians should be hesitant to disclose prodromal PD, regardless of the patient’s wish to be informed [21]. Finally, diagnosing healthy individuals based on risk factors brings along ethical dilemmas, which requires adequate information provision about the reasons for risk assessment and potential consequences of knowing ones risk [22].

The relative importance of increasing the treatment effect was highest if the effectiveness increased from gaining 1 year to gaining 5 years. This suggest that disease-modifying treatments in prodromal PD that delay the diagnosis by only 1 year do not have much value for professionals, but this changes rapidly when treatments would delay time to diagnoses by 5 years or more. Participants were willing to accept a higher risk of severe side-effects if the treatment effect increased by delaying time to diagnosis with an additional year, especially when the delay in time to the diagnoses increased by an additional year between 1 and 5 years. That an effect increase from, for example, gaining 3 to 4 years is valued as more important than an effect increase from, for example, gaining 15 to 16 years can be explained by temporal discounting. Temporal discounting is the tendency of people to give greater utility to things that might happen in the near future compared to the late future [23].

This is the first study that analyzes decision-making for disease-modifying treatments in prodromal PD. It also illustrates how a DCE can be a useful method to gain a deeper understanding of the preferences for choosing interventions in (prodromal) PD. As they are relatively quick and inexpensive survey instruments, DCEs present various advantages for informing decision-making. We performed a thorough pilot phase to define our treatment attributes and levels, and we presented the results of this pilot in the Supplementary Material.

This study also has some limitations that impact the extent to which our findings predict preferences for future preventive treatment in PD. First, we used hypothetical treatment descriptions because disease-modifying treatments are not yet available in the prodromal phase of PD. It is uncertain to what extent the attributes and levels to describe the treatments are a reflection of actual disease-modifying treatments that might become available in the future. Second, respondents were from the Netherlands only, and therefore the results of the study are only representative for the Netherlands. Results in this study need to be verified on a larger scale and in an international context. Finally, a DCE is a stated preference method, and it is known that stated preferences can differ from revealed preferences [15]. In a DCE, treatment choice is based on the attributes and levels presented in the study, which is generally a simplification of real life clinical decision making.

In conclusion, this study provides first insights into factors which will influence decision making by professionals for starting an intervention in the prodromal phase of PD. When disease-modifying treatments would become available, this information can be used for a successful implementation.

## Supplementary Material

Supplementary Material
