# Supplementary material for: Professionals’ Treatment Preferences in the Prodromal Phase of Parkinson’s Disease: A Discrete Choice Experiment
Source: J Parkinsons Dis. 2022 Jul 8;12(5):1655–64. doi: 10.3233/JPD-223208 (PMC9398060; doi:10.3233/JPD-223208)
Supplement: Supplementary Material [file jpd-12-jpd223208-s001.pdf]

Supplementary Material

Professionals' Treatment Preferences in the Prodromal Phase of Parkinson's Disease: A Discrete Choice Experiment

Table of contents

METHODS 2

Step 1: Research question 2

Step 2: Attributes and levels 2

Literature review 3

Semi-structured interviews 3

Selection of attributes and levels 4

Step 3. Construction of tasks 5

Step 4: Experimental design 5

Step 5: Preference elicitation 6

Step 6: Instrument design 6

Step 7. Data collection plan 7

Step 8. Statistical analysis 7

RESULTS 9

Literature review 9

Subanalysis only neurologists 11

Logistic regression model opt-in/out question 12

Qualifying question lifestyle 13

REFERENCES 14

METHODS

Preferences regarding treatment in the prodromal phase in PD were elicited using an online survey which contained a Discrete Choice Experiment (DCE). In a DCE, respondents answer multiple preference elicitation tasks. Respondents are asked to indicate their preference for one of two or more hypothetical profiles, which are descriptions of possible interventions. Profiles consist of different attributes, which are the characteristics of the interventions that are compared (e.g., risk on side effects), and levels, which are descriptions of the range of potential outcomes for each attribute (e.g., 5% chance of a side-effect). Analysis of the stated preferences of respondents enables researchers to assess the extent to which the attributes of the intervention drive preferences, and the trade-offs respondents are willing to make between different attribute outcomes.

We adopted a systematic approach, consisting of multiple, consecutive steps in the process of constructing a DCE based on several articles on DCE experimental designs [1-3]. Also, we applied an existing checklist for properly designing a DCE [4], including: 1) research question; 2) attributes and levels; 3) construction of tasks; 4) experimental design; 5) preference elicitation; 6) instrument design; 7) data collection plan; 8) statistical analyses; 9) results and conclusions; and 10) study presentation.

Step 1: Research question

The aim of this study was to explore preferences of professionals regarding the choice for disease-modifying treatment in the prodromal stage of PD. We formulated two underlying research questions: First, what preferences do professionals have for different treatment characteristics of disease-modifying treatment in the prodromal stage of PD? Second, what preferences do professionals have for actual prescribing disease-modifying treatment to patients in the prodromal stage of PD?

Step 2: Attributes and levels

To select the attributes and levels, we first explored which factors influence decision making of neurologists on starting disease-modifying treatment in the prodromal phase of PD. A literature review was conducted, and subsequently, in-depth semi-structured interviews with neurologists.

Literature review

A literature review was conducted by one member of the research team (WH) in the PubMed database (searched performed in June 2020). No research exists on factors influencing the neurologists' decision-making in the prodromal phase of PD. Therefore, we first looked at which treatment factors neurologists would value in PD treatment in general. The search terms *((physician) AND (Parkinson Disease) AND (treatment) AND (preferences))* yielded 38 results, of which 12 were selected based on title and abstract [5-15]. Inclusion criteria were: 1) study evaluates decision-making in PD treatment options and 2) study includes neurologists in the target population. No exclusion criteria were defined. Potential factors influencing treatment decision-making were included in a list for attribute selection. Secondly, we looked at DCE studies focusing on decision-making on treatment characteristics, in general. An advantage of focusing on DCEs was that it provided insight into how other studies operationalize their attributes and their corresponding levels. The search terms *("therapy" [Subheading]) AND ("discrete choice experiment"[All Fields] OR "DCE"[All Fields]) NOT ("DCE-MRI" OR "DCE-CT" OR "contrast-enhanced" OR "MRI")* yielded 681 results, of which 33 articles were selected for the review [16-43]. Inclusion criteria were: 1) study uses a DCE that is based on a choice between two profiles, 2) study evaluates trade-offs in treatment characteristics, and 3) study includes medical professionals in their target population. No exclusion criteria were defined. We summarized all articles in a literature data extraction spreadsheet and created a list of factors influencing the decision-making of physicians on starting a new treatment (Supplementary Figure 1).

Semi-structured interviews

We subsequently conducted in-depth, semi-structured interviews with neurologists, all specialized in movement disorders. Semi-structured interviews allow for a deeper insight into the opinion of neurologists on factors that would influence their decision-making on starting a potential treatment for patients in the prodromal phase of PD. A key advantage of using semi-structured interviews is that it provides a loose structure to explore the attitude of neurologists and moreover, it gives the flexibility to explore unexpected findings in more detail. Eight potential participants were invited by email including an information letter, and written informed consent was obtained prior to the interview. In total, five neurologists and one neurologists who is an expert in prodromal PD research agreed to participate. During the interviews, we used an interview guide

with initial topics and questions focusing on patient- and treatment factors, in addition to potential follow-up probing questions. We composed the interview guide based on the literature study and discussions within the research team. The interviews lasted maximally 45 minutes and were conducted by phone. The interviews were audio-recorded using a separate recorder.

We pseudonymized and transcribed the audio recordings verbatim (using F4transkript, version 3.1). Then, the transcripts were coded one by one to allow for the implementation of new insights in the following interviews (using Atlas.ti, version 8.4). Codes were derived deductively using the pre-defined categories from the interview guide, but also inductively using open encoding to allow for unexpected categories in the analysis. In the beginning, two researchers independently coded the first two interviews (WH and LH). After these two interviews, the two researchers discussed their codes to make sure that they interpreted the data equally. Differences were resolved in group discussion and a thematic framework was developed, based on the agreed set of themes and categories. Then WH applied this framework to the last four interviews. Dilemmas were resolved in group discussion. Following the last coding, we charted and summarized the acquired qualitative data in a spreadsheet (Excel) by category and by participant. We used this chart to help to interpret the data, facilitating analyses both within categories and respondents (results of the interviews in Supplementary Figure 2).

Selection of attributes and levels

From the interviews, the following five treatment attributes were selected: gained years to diagnosis (to address treatment effect), risk on mild side-effects, risk on severe side-effects, route of administration, and annual costs. Improvement in health in the prodromal phase of PD was defined as slowing down disease progression, which was operationalized into the attribute 'delay in years to diagnosis'. Risk of side-effects was operationalized in two different attributes: risk of mild side-effects and risk of severe side-effects. Cost of treatment was defined as annual costs. Based on the interviews, a fifth treatment attributed was added: route of administration. We based the initial attribute selection on treatment attributes most frequently used in the literature. Subsequently, the interviews were used to determine how these initial treatment attributes would relate to hypothetical treatments in the prodromal phase of PD. Since there is no defined strategy for attribute and level selection, we selected attributes based on which factors were described as important in the literature and were valued most important in the interviews with neurologists. For

the level selection, we selected values that were realistic and also covered a sufficiently broad spectrum, using existing prescribing information, literature, and the researchers' opinion. It is not advised to include more than six attributes in a DCE, with a maximum of four levels each, since the design would otherwise be too complicated and lose efficiency [1, 44].

Step 3. Construction of tasks

Conceptualizing the choice process was done based on group consensus. We opted for a generic choice-based DCE design with two full profiles of alternatives, because we aimed to simulate future decision-making between two disease-modifying treatments. We focussed on pharmacological disease-modifying treatments rather than also including lifestyle treatments, since this would complicate the attribute and level selection. No opt-out question was included to force participants to make a decision, but we rather decided to include a secondary question following every choice set asking participants if they, in clinical practice, would give their chosen treatment to these patients or not.

Step 4: Experimental design

The experimental design was designed in the software R. First, the full factorial design was estimated. Then, choice tasks with dominant profiles of overlapping attribute-levels were removed from the full set. No restrictions on implausible attribute level combinations were set. Then, 1000 random sets of 32 choice tasks were drawn from the full set, and the set with the highest D-efficiency was selected. This resulted in a designs that was nearly balanced and nearly orthogonal. To reduce cognitive difficulty the design was blocked into four blocks of eight choice sets. Questions within each block were randomized. Each respondent was randomized to receive 1 block of choice sets for a high risk profile and a different block of choice sets for a moderate risk profile. Thus, each respondent was ask to choose their preferred treatment for in total 16 choice sets.

Following the construction of the survey, we tested the first draft survey with eight neurologists to check the attribute and level coverage, the understanding and complexity of the survey, and also the length of the survey. To do this, we invited ten neurologists in the professional network of BP by mail for a phone- or video call, of which four neurologists also participated in the interview

study. We presented neurologists with the DCE, while the researcher probed them to think aloud and provide feedback. Provided feedback was then evaluated and incorporated into the DCE.

Step 5: Preference elicitation

The survey started with an explanation of the preference elicitation task, the definitions of the attributes and a definition of a high (80%) and moderate (30%) risk profile. Also, we included two qualifying questions. The first qualifying question was a multiple option question on which other important attributes would influence the participant's decision to start an intervention in the prodromal phase of PD. The second qualifying question was a multiple choice question on the participants' opinion on lifestyle advice in the prodromal phase of PD, while there is evidence that lifestyle interventions might also be beneficial in the prodromal phase of PD [45, 46]. We chose not to include both lifestyle and non-labeled pharmacological treatments in one DCE. Including lifestyle as a labelled treatment choice in the DCE would carry the risk of participants choosing for or against this option based on their prior beliefs and not on the presented attributes and levels. Moreover, during the interviews most neurologists indicated that they would always consider lifestyle treatment. Instead, we included this follow-up question on lifestyle advice in the third part of the online survey (Supplementary Table 4).

Step 6: Instrument design

First, participants were presented with an introduction to inform them on the goal and structure of the study and a set of questions collecting participant characteristics (e.g., gender, type of hospital, years of experience as a neurologist, knowledge of the prodromal phase of PD). This was followed by the 16 choice sets where participants had to answer which treatment (treatment A or treatment B) they would give to 1) a patient with a high risk (80%) of being in the prodromal phase or 2) a patient with a medium risk (30%) of being in the prodromal phase. Following every choice set, participants also had to answer if they, in clinical practice, would give their chosen treatment to these patients or not. Participants could constantly view a description of all the attributes to enhance understanding of the attributes and levels. We added a dominant question to validate the internal validity of the survey, which allows for the exclusion of participants in the analysis when they would not choose the dominant alternative. At the end of the survey, we included one multiple-choice question to ask participants what their opinion was on the role of lifestyle

interventions as a disease-modifying therapy for PD (Supplementary Table 4), and one open question on if there were any missing attributes or levels in the experiment (main manuscript).

Step 7. Data collection plan

Different methods exist to calculate sample size in DCEs. Johnson and Orme suggest the following rule of thumb to calculate the required sample size: $N > \frac{500 \times largest\ number\ of\ levels}{number\ of\ choice\ sets \times number\ of\ alternatives}$ [47]. With the proposed DCE with 16 choice sets per participants, two alternatives per choice set and a maximum of four levels per attribute, the required sample size was 63 according to this rule of thumb. However, as others propose at least 100 respondents [47], we aimed for a sample size between 63 and 100.

We built the DCE in a web-based survey tool (LimeSurvey) to allow for easy distribution. The study protocol was approved by the Medical Ethics Committee of the Radboud university medical center and registered as 2020-6627. All participants gave written informed consent prior to the study.

Step 8. Statistical analysis

The choice sets were analyzed using an effect-coded conditional logit model [48] using Cox regression in SPSS Statistics 25 (IBM). Only those participants who selected the dominant treatment option in the internal validity question, were included in the analysis. The conditional logit model relates the probability of choice between two or more alternatives to the characteristics of the presented attribute levels. McFadden has shown that the conditional logit model is consistent with random utility theory [49]. Random utility theory assumes that the utility of an alternative is a function of characteristics (attribute levels) of the alternative and that individuals will choose the alternative that maximizes their utility (U). The following equation is used to calculate the utility of the treatment alternatives: $U = \beta Years\ gained\ before\ diagnosis + \beta Mild\ side\ effects + \beta Severe\ side\ effects + \beta Route\ of\ administration + \beta Costs + \varepsilon$, where β is the coefficient of the corresponding attribute, indicating the impact of that attribute on choosing a specific treatment and ε is the error term [48, 50]. The estimated β-coefficient is a preference weight, and it represents the relative contribution of the attribute level to the utility that respondents assign to an alternative (part-worth utility estimate). Because effect-coding was used, the estimated preference weights for the hypothetical treatment attribute are relative to the mean effect,

normalized at zero. The signs of the β-coefficients indicate whether the attribute has a negative or positive effect on utility. An odds ratio can be calculated taking the exponent of β. Differences between part-worth utility for different levels indicate the relative importance of moving from one level of an attribute to an adjacent level of that attribute: the greater the difference, the more important the change from one level to the next (within-attribute). The relative importance of each attribute (between-attribute) is a measure of the relative influence of the change from worst to best outcome on each attribute on the overall utility of the treatment. Thus, it is a measure of the relative importance of the attribute in choosing between treatments. The relative importance of each attribute is calculated by taking the range in part-worth utility estimate for the best and worst levels of each attribute, and divide it by the sum of the range in part-worth utility of all other attributes. The following formula is used: $W_{attribute_i} = \frac{max\beta_i - min\beta_i}{\Sigma_{j(max\beta_j - min\beta_j)}}$. Predefined subgroup analysis included analysis from data from neurologists only (Supplementary Tables 1 and 2).

A logistic regression model was used to analyze how individual treatment attributes and risk profile influenced the choice to discuss the treatment with the patient in daily practice, using only the chosen treatment from each choice set (Supplementary Table 3). The dependent variable was the choice: opt-in (yes) or opt-out (no). Independent variables included the treatment attributes and levels described above, and the risk profile.

The marginal rate of substitution represents the rate at which respondents are willing to trade off among the attributes. We calculated the willingness to accept an increase in risk of severe side-effects between the interval of 1-5% to gain an additional year in time to diagnosis, the two most highly valued attributes. The willingness to accept an increase in risk of severe-side effects to gain an additional life year in time to diagnosis was calculating by dividing the utility gain for gaining 1 additional year in time to diagnosis by the utility loss of 1% increased risk of severe side effects. We calculated the utility loss from of an additional 1% risk of severe side effects between the levels 1% and 5%, because the difference in utility in this attribute was greatest between those levels. We separately calculated the utility gain for gaining 1 additional year in time to diagnoses between the levels 1 and 5 year, 5 and 10 years, and 10 and 20 years.

RESULTS

Literature review

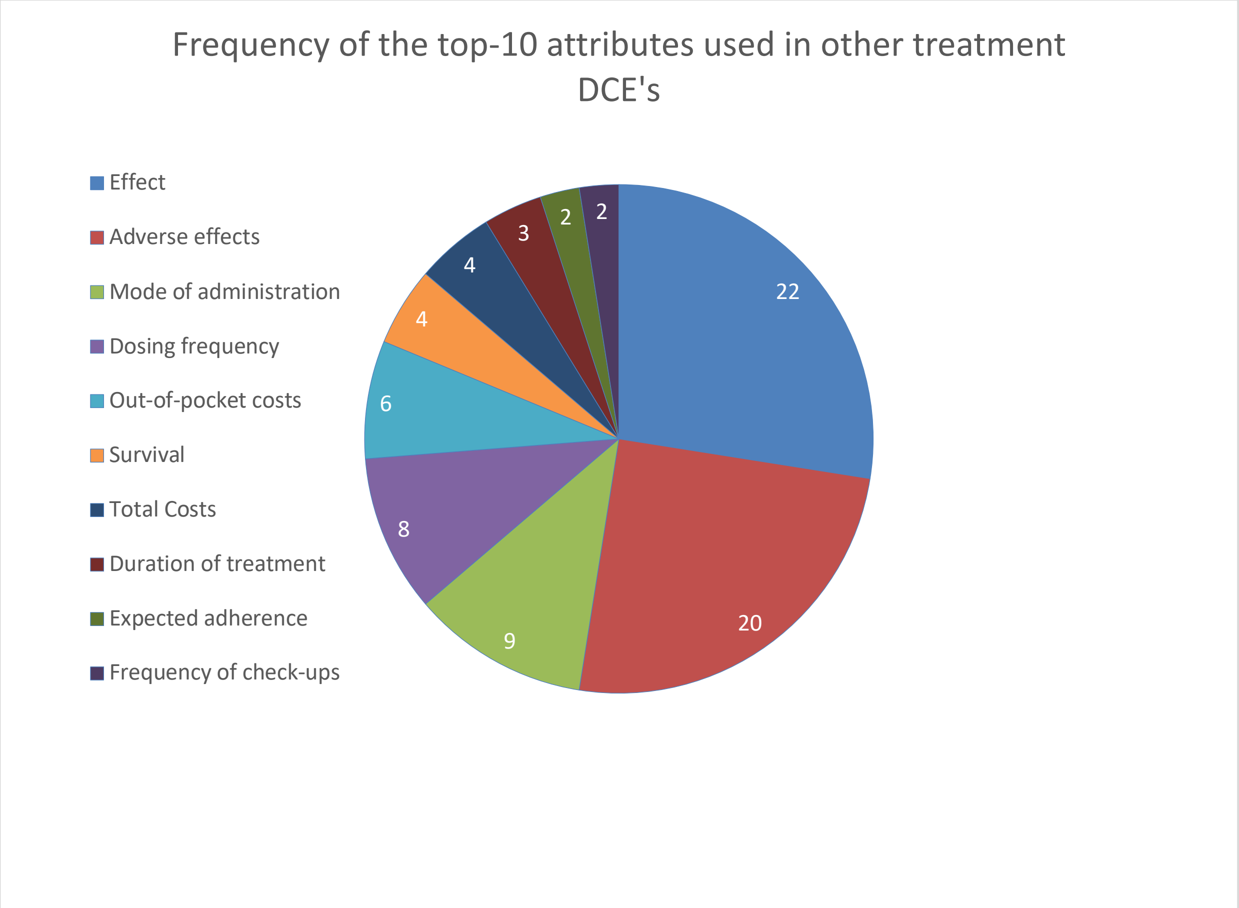

Supplementary Figure 1. Frequency of the top-10 attributes used in other healthcare treatment DCEs. Emerging out of the DCE articles in the literature study [16-43].

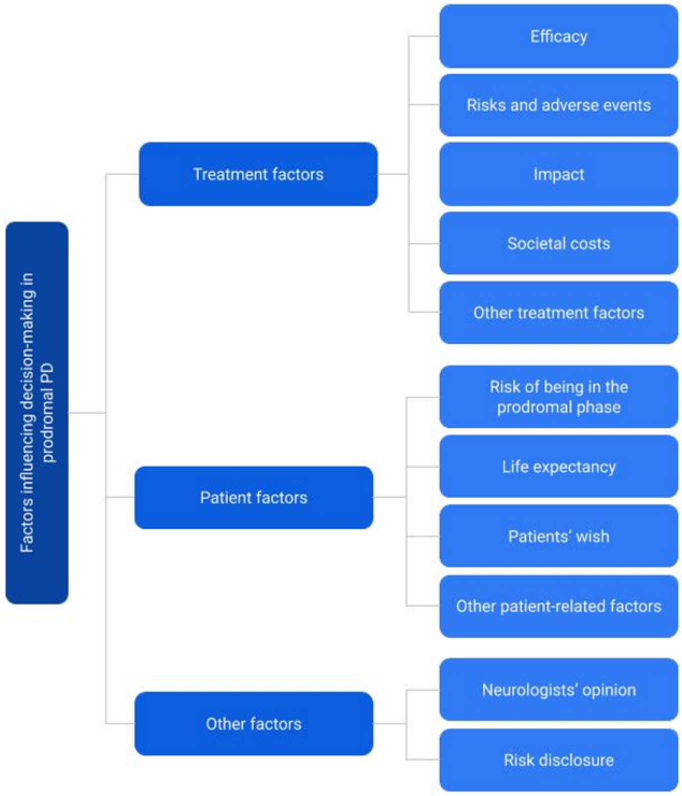

Supplementary Figure 2. Overarching themes and subthemes, emerging from the semi-structured interviews.

Subanalysis only neurologists

	HIGH RISK (80%)					MODERATE RISK (30%)				
	β	SE	p	Exp(β)	95% CI Exp(β)	β	SE	p	Exp(β)	95% CI Exp(β)
**EFFECT**			0.000					0.000		
20 years gained	1.716	0.268	0.000	5.561	3.291-9.398	1.119	0.201	0.000	3.060	2.062-4.541
10 years gained	0.904	0.163	0.000	2.469	1.792-3.401	1.037	0.161	0.000	2.821	2.059-3.865
5 years gained	-0.363	0.193	0.060	0.696	0.477-1.016	-0.350	0.182	0.054	0.705	0.494-1.006
1 year gained	-2.257	0.375	0.000	0.105	0.050-0.218	-1.806	0.251	0.000	0.164	0.100-0.269
**MILD SIDE-EFFECTS**			0.006					0.000		
20% risk	0.624	0.211	0.003	1.866	1.234-2.822	0.746	0.203	0.000	2.108	1.416-3.138
40% risk	-0.118	0.165	0.475	0.889	0.644-1.228	-0.179	0.155	0.247	0.836	0.617-1.132
60% risk	-0.506	0.176	0.004	0.603	0.427-0.851	-0.567	0.161	0.000	0.567	0.414-0.777
**SEVERE SIDE-EFFECTS**			0.000					0.000		
0.01% risk	0.908	0.247	0.000	2.479	1.528-4.020	0.853	0.217	0.000	2.346	1.532-3.592
0.1% risk	0.571	0.250	0.022	1.770	1.085-2.890	0.623	0.207	0.003	1.864	1.243-2.795
1% risk	0.136	0.179	0.449	1.145	0.806-1.628	0.123	0.157	0.434	1.131	0.831-1.540
5% risk	-1.615	0.292	0.000	0.199	0.112-0.352	-1.599	0.226	0.000	0.202	0.130-0.315
**ROUTE OF ADMINISTRATION**			0.006					0.075		
Orally daily	-0.034	0.318	0.915	0.967	0.518-1.803	-0.412	0.230	0.073	0.662	0.422-1.039
Orally 3 times a day	-0.110	0.241	0.648	0.896	0.559-1.437	0.006	0.179	0.971	1.007	0.708-1.430
Weekly injection	-0.409	0.155	0.008	0.665	0.491-0.900	-0.054	0.149	0.717	0.947	0.707-1.269
Six-monthly injection	0.552	0.225	0.014	1.737	1.117-2.700	0.460	0.179	0.010	1.584	1.116-2.249
**ANNUAL COSTS**			0.001					0.001		
€ 100	0.532	0.187	0.004	1.703	1.180-2.459	0.285	0.151	0.059	1.330	0.989-1.789
€ 1000	0.230	0.138	0.097	1.258	0.959-1.651	0.278	0.140	0.047	1.321	1.004-1.737
€ 10.000	-0.762	0.197	0.000	0.467	0.317-0.687	-0.563	0.146	0.000	0.569	0.428-0.758

Supplementary Table 1. Estimated preference weights for subanalysis using neurologists only (n=63). Effect-coding was used for categorical variable coding: the estimated preference weights for the hypothetical treatment attribute are relative to the mean effect, normalized at zero. The signs of the β-coefficients indicate whether the attribute has a negative or positive effect on utility. The Exp(β) represents the odds ratio.

Logistic regression model opt-in/out question

	β	S.E.	Sign.	Exp(β)	95% CI Exp(β)
**Effect**			0.005		
Effect(1) – 20y	0.483	0.226	0.033	1.621	1.040-2.526
Effect(2) – 10y	0.363	0.219	0.097	1.438	0.937-2.207
Effect(3) – 5y	-0.176	0.253	0.489	0.839	0.511-1.379
**Mild side-effects**			0.001		
Mild SE (1) -20%	0.338	0.180	0.061	1.402	0.985-1.995
Mild SE (2) -40%	-0.344	0.169	0.042	0.709	0.509-.988
**Severe side-effects**			0.943		
Severe SE(1) -0.01%	.014	0.234	0.952	1.014	0.641-1.604
Severe SE(2)-0.1%	-0.067	0.238	0.777	0.935	0.586-1.490
Severe SE(3)-1%	0.050	0.244	0.837	1.051	0.652-1.696
**Route of administration**			0.000		
RoA(1)-orally daily	-0.900	0.211	0.000	0.407	0.269-.615
RoA(2)- orally 3x a day	-0.987	0.179	0.000	0.373	0.263-.529
RoA(3)- weekly injection	-1.198	0.186	0.000	0.302	0.209-.435
**Annual costs**			0.000		
Costs(1) -€100	1.021	0.185	0.000	2.775	1.933-3.985
Costs(2)- €1000	0.875	0.192	0.000	2.400	1.649-3.493
**Riskprofile**					
Riskprofile – high risk	1.427	0.140	0.000	4.167	3.168-5.481

Supplementary Table 3. Logistic regression model. Dependent variable was opt-in (participant would discuss that treatment in daily practice). Independent variables included treatment attributes and riskprofile. The last level in each category was the omitted variable. Treatment attributes: Effect: years gained until diagnosis resp 20y, 10y, 5y, 1y; Mild SE: risk on mild side-effects resp 20%, 40%, 60%; Severe SE: risk on severe side-effects resp 0.01, 0.1%, 1%, 5%. RoA: Route of administration resp orally daily, orally 3x a day, weekly injection, six-monthly injection. Annual costs resp. €100, €1000, €10.000. Riskprofile: high risk and moderate risk profile.

Qualifying question lifestyle

	N
I think there is no effect	17 (20.7%)
I think there is an effect but patients will not adhere	19 (23.2%)
I think there is an effect and patients will adhere	35 (42.7%)
I think there is an effect and that it is better than pharmacological treatment	11 (13.4%)
Total	82 (100%)

Supplementary Table 4. Answers on qualifying question ''What do you think of lifestyle advice in the prodromal phase of Parkinson's disease?''

REFERENCES

[1] Lancsar E, Louviere J. Conducting discrete choice experiments to inform healthcare decision making: a user's guide. Pharmacoeconomics. 2008;26(8):661-77.

[2] WHO. How to conduct a discrete choice experiment for health workforce recruitment and retention in remote and rural areas : a user guide with case studies. Washington, DC: International Bank for Reconstruction and Development/World Bank; 2013.

[3] Bridges JFP, Hauber AB, Marshall D, Lloyd A, Prosser LA, Regier DA, et al. Conjoint Analysis Applications in Health—a Checklist: A Report of the ISPOR Good Research Practices for Conjoint Analysis Task Force. Value in Health. 2011;14(4):403-13.

[4] Bridges JF, Hauber AB, Marshall D, Lloyd A, Prosser LA, Regier DA, et al. Conjoint analysis applications in health--a checklist: a report of the ISPOR Good Research Practices for Conjoint Analysis Task Force. Value Health. 2011;14(4):403-13.

[5] Fargel M, Grobe B, Oesterle E, Hastedt C, Rupp M. Treatment of Parkinson's disease: a survey of patients and neurologists. Clin Drug Investig. 2007;27(3):207-18.

[6] Gallagher DA, Schrag A. Impact of newer pharmacological treatments on quality of life in patients with Parkinson's disease. CNS Drugs. 2008;22(7):563-86.

[7] Kishore A, Snow BJ. Drug management of Parkinson's disease. Can Fam Physician. 1996;42:946-52.

[8] Larisch A, Oertel WH, Eggert K. Attitudes and barriers to clinical practice guidelines in general and to the guideline on Parkinson's disease. A National Survey of German neurologists in private practice. J Neurol. 2009;256(10):1681-8.

[9] Löhle M, Ramberg CJ, Reichmann H, Schapira AH. Early versus delayed initiation of pharmacotherapy in Parkinson's disease. Drugs. 2014;74(6):645-57.

[10] Morris JG. The drug treatment of Parkinson's disease. Aust Fam Physician. 1984;13(5 Suppl):6-8, 11.

[11] Nijhuis FA, van Heek J, Bloem BR, Post B, Faber MJ. Choosing an Advanced Therapy in Parkinson's Disease; is it an Evidence-Based Decision in Current Practice? J Parkinsons Dis. 2016;6(3):533-43.

[12] Nisenzon AN, Robinson ME, Bowers D, Banou E, Malaty I, Okun MS. Measurement of patient-centered outcomes in Parkinson's disease: what do patients really want from their treatment? Parkinsonism Relat Disord. 2011;17(2):89-94.

[13] Tarsy D. Initial treatment of Parkinson's disease. Curr Treat Options Neurol. 2006;8(3):224-35.

[14] Weernink MG, van Til JA, van Vugt JP, Movig KL, Groothuis-Oudshoorn CG, MJ IJ. Involving Patients in Weighting Benefits and Harms of Treatment in Parkinson's Disease. PLoS One. 2016;11(8):e0160771.

[15] Wüllner U, Fuchs G, Reketat N, Randerath O, Kassubek J. Requirements for Parkinson's disease pharmacotherapy from the patients' perspective: a questionnaire-based survey. Curr Med Res Opin. 2012;28(7):1239-46.

[16] Arellano J, Hauber AB, Mohamed AF, Gonzalez JM, Collins H, Hechmati G, et al. Physicians' preferences for bone metastases drug therapy in the United States. Value Health. 2015;18(1):78-83.

[17] Ashcroft DM, Seston E, Griffiths CE. Trade-offs between the benefits and risks of drug treatment for psoriasis: a discrete choice experiment with U.K. dermatologists. Br J Dermatol. 2006;155(6):1236-41.

[18] Baji P, Gulacsi L, Lovasz BD, Golovics PA, Brodszky V, Pentek M, et al. Treatment preferences of originator versus biosimilar drugs in Crohn's disease; discrete choice experiment among gastroenterologists. Scand J Gastroenterol. 2016;51(1):22-7.

[19] Benjamin L, Cotté FE, Philippe C, Mercier F, Bachelot T, Vidal-Trécan G. Physicians' preferences for prescribing oral and intravenous anticancer drugs: a Discrete Choice Experiment. Eur J Cancer. 2012;48(6):912-20.

[20] Berchi C, Degieux P, Halhol H, Danel B, Bennani M, Philippe C. Impact of falling reimbursement rates on physician preferences regarding drug therapy for osteoarthritis using a discrete choice experiment. Int J Pharm Pract. 2016;24(2):114-22.

[21] Bolt T, Mahlich J, Nakamura Y, Nakayama M. Hematologists' Preferences for First-line Therapy Characteristics for Multiple Myeloma in Japan: Attribute Rating and Discrete Choice Experiment. Clin Ther. 2018;40(2):296-308.e2.

[22] Bröckelmann PJ, McMullen S, Wilson JB, Mueller K, Goring S, Stamatoullas A, et al. Patient and physician preferences for first-line treatment of classical Hodgkin lymphoma in Germany, France and the United Kingdom. Br J Haematol. 2019;184(2):202-14.

[23] Byun J-H, Kwon S-H, Lee J-E, Cheon J-E, Jang E-J, Lee E-K. Comparison of benefit-risk preferences of patients and physicians regarding cyclooxygenase-2 inhibitors using discrete choice experiments. Patient Prefer Adherence. 2016;10:641-50.

[24] Carlsen B, Hole AR, Kolstad JR, Norheim OF. When you can't have the cake and eat it too: a study of medical doctors' priorities in complex choice situations. Soc Sci Med. 2012;75(11):1964-73.

[25] De Brún A, Flynn D, Ternent L, Price CI, Rodgers H, Ford GA, et al. Factors that influence clinicians' decisions to offer intravenous alteplase in acute ischemic stroke patients with uncertain treatment indication: Results of a discrete choice experiment. Int J Stroke. 2018;13(1):74-82.

[26] Diorio C, Tomlinson D, Boydell KM, Regier DA, Ethier MC, Alli A, et al. Attitudes toward infection prophylaxis in pediatric oncology: a qualitative approach. PLoS One. 2012;7(10):e47815.

[27] Ettinger AB, Carter JA, Rajagopalan K. Patient versus neurologist preferences: A discrete choice experiment for antiepileptic drug therapies. Epilepsy Behav. 2018;80:247-53.

[28] Feldman SR, Regnier SA, Chirilov A, Hey F, Gilloteau I, Cella D. Patient-reported outcomes are important elements of psoriasis treatment decision making: A discrete choice experiment survey of dermatologists in the United States. J Am Acad Dermatol. 2019;80(6):1650-7.

[29] González JM, Doan J, Gebben DJ, Boeri M, Fishman M. Comparing the Relative Importance of Attributes of Metastatic Renal Cell Carcinoma Treatments to Patients and Physicians in the United States: A Discrete-Choice Experiment. Pharmacoeconomics. 2018;36(8):973-86.

[30] Hifinger M, Hiligsmann M, Ramiro S, Watson V, Severens JL, Fautrel B, et al. Economic considerations and patients' preferences affect treatment selection for patients with rheumatoid arthritis: a discrete choice experiment among European rheumatologists. Ann Rheum Dis. 2017;76(1):126-32.

[31] Ivanova J, Hess LM, Garcia-Horton V, Graham S, Liu X, Zhu Y, et al. Patient and Oncologist Preferences for the Treatment of Adults with Advanced Soft Tissue Sarcoma: A Discrete Choice Experiment. Patient. 2019;12(4):393-404.

[32] Landfeldt E, Eriksson J, Ireland S, Musingarimi P, Jackson C, Tweats E, et al. Patient, physician, and general population preferences for treatment characteristics in relapsed or refractory chronic lymphocytic leukemia: A conjoint analysis. Leuk Res. 2016;40:17-23.

[33] Liu FX, Witt EA, Ebbinghaus S, DiBonaventura Beyer G, Basurto E, Joseph RW. Patient and Oncology Nurse Preferences for the Treatment Options in Advanced Melanoma: A Discrete Choice Experiment. Cancer Nurs. 2019;42(1):E52-e9.

[34] Lock J, de Bekker-Grob EW, Urhan G, Peters M, Meijer K, Brons P, et al. Facilitating the implementation of pharmacokinetic-guided dosing of prophylaxis in haemophilia care by discrete choice experiment. Haemophilia. 2016;22(1):e1-e10.

[35] Mantovani LG, Monzini MS, Mannucci PM, Scalone L, Villa M, Gringeri A. Differences between patients', physicians' and pharmacists' preferences for treatment products in haemophilia: a discrete choice experiment. Haemophilia. 2005;11(6):589-97.

[36] McMullen S, Hess LM, Kim ES, Levy B, Mohamed M, Waterhouse D, et al. Treatment Decisions for Advanced Non-Squamous Non-Small Cell Lung Cancer: Patient and Physician Perspectives on Maintenance Therapy. Patient. 2019;12(2):223-33.

[37] Morgan JL, Walters SJ, Collins K, Robinson TG, Cheung KL, Audisio R, et al. What influences healthcare professionals' treatment preferences for older women with operable breast cancer? An application of the discrete choice experiment. Eur J Surg Oncol. 2017;43(7):1282-7.

[38] Mühlbacher AC, Bethge S. Reduce mortality risk above all else: a discrete-choice experiment in acute coronary syndrome patients. Pharmacoeconomics. 2015;33(1):71-81.

[39] Muhlbacher AC, Nubling M. Analysis of physicians' perspectives versus patients' preferences: direct assessment and discrete choice experiments in the therapy of multiple myeloma. Eur J Health Econ. 2011;12(3):193-203.

[40] Nakayama M, Kobayashi H, Okazaki M, Imanaka K, Yoshizawa K, Mahlich J. Patient Preferences and Urologist Judgments on Prostate Cancer Therapy in Japan. Am J Mens Health. 2018;12(4):1094-101.

[41] Pacou M, Basso F, Gore C, Hass B, Taieb V, Cognet M, et al. Patient and physician preferences for the treatment of chronic hepatitis C virus infections: does the perspective matter? Eur J Gastroenterol Hepatol. 2015;27(9):1063-8.

[42] Park MH, Jo C, Bae EY, Lee EK. A comparison of preferences of targeted therapy for metastatic renal cell carcinoma between the patient group and health care professional group in South Korea. Value Health. 2012;15(6):933-9.

[43] Poulos C, Reed Johnson F, Krishnarajah G, Anonychuk A, Misurski D. Pediatricians' preferences for infant meningococcal vaccination. Value Health. 2015;18(1):67-77.

[44] Reed Johnson F, Lancsar E, Marshall D, Kilambi V, Mühlbacher A, Regier DA, et al. Constructing experimental designs for discrete-choice experiments: report of the ISPOR Conjoint Analysis Experimental Design Good Research Practices Task Force. Value Health. 2013;16(1):3-13.

[45] Fang X, Han D, Cheng Q, Zhang P, Zhao C, Min J, et al. Association of Levels of Physical Activity With Risk of Parkinson Disease: A Systematic Review and Meta-analysis. JAMA Netw Open. 2018;1(5):e182421.

[46] Maraki MI, Yannakoulia M, Stamelou M, Stefanis L, Xiromerisiou G, Kosmidis MH, et al. Mediterranean diet adherence is related to reduced probability of prodromal Parkinson's disease. Mov Disord. 2019;34(1):48-57.

[47] de Bekker-Grob EW, Donkers B, Jonker MF, Stolk EA. Sample Size Requirements for Discrete-Choice Experiments in Healthcare: a Practical Guide. Patient. 2015;8(5):373-84.

[48] Hauber AB, González JM, Groothuis-Oudshoorn CG, Prior T, Marshall DA, Cunningham C, et al. Statistical Methods for the Analysis of Discrete Choice Experiments: A Report of the ISPOR Conjoint Analysis Good Research Practices Task Force. Value Health. 2016;19(4):300-15.

[49] McFadden D. Conditional logit analysis of qualitative choice behavior. Zarembka P, Ed, Frontiers in Econometrics Academic Press. 1973:105-42.

[50] Benjamin L, Cotté F-E, Philippe C, Mercier F, Bachelot T, Vidal-Trécan G. Physicians' preferences for prescribing oral and intravenous anticancer drugs: A Discrete Choice Experiment. European Journal of Cancer. 2012;48(6):912-20.
